# Bovine Papillomavirus Type 2 (BPV-2) E5 Oncoprotein Binds to the Subunit D of the V_1_-ATPase Proton Pump in Naturally Occurring Urothelial Tumors of the Urinary Bladder of Cattle

**DOI:** 10.1371/journal.pone.0088860

**Published:** 2014-02-24

**Authors:** Sante Roperto, Valeria Russo, Giuseppe Borzacchiello, Chiara Urraro, Roberta Lucà, Iolanda Esposito, Marita Georgia Riccardi, Cinzia Raso, Marco Gaspari, Dora Maria Ceccarelli, Rocco Galasso, Franco Roperto

**Affiliations:** 1 Dipartimento di Medicina Veterinaria e Produzioni Animali, Settore Malattie Infettive, Università di Napoli Federico II, Napoli, Italia; 2 Dipartimento di Medicina Veterinaria e Produzioni Animali, Settore Patologia Generale, Università di Napoli Federico II, Napoli, Italia; 3 Dipartimento di Medicina Clinica e Sperimentale, Università di Catanzaro “Magna Graecia”, Catanzaro, Italia; 4 Unit of clinical epidemiology, biostatistic and cancer registry, IRCCS CROB, Rionero in Vulture (Potenza), Italia; 5 Dipartimento di Biologia, Università di Napoli Federico II, Napoli, Italia; National Institute of Health - National Cancer Institute, United States of America

## Abstract

**Background:**

Active infection by bovine papillomavirus type 2 (BPV-2) was documented for fifteen urinary bladder tumors in cattle. Two were diagnosed as papillary urothelial neoplasm of low malignant potential (PUNLMP), nine as papillary and four as invasive urothelial cancers.

**Methods and Findings:**

In all cancer samples, PCR analysis revealed a BPV-2-specific 503 bp DNA fragment. E5 protein, the major oncoprotein of the virus, was shown both by immunoprecipitation and immunohistochemical analysis. E5 was found to bind to the activated (phosphorylated) form of the platelet derived growth factor β receptor. PDGFβR immunoprecipitation from bladder tumor samples and from normal bladder tissue used as control revealed a protein band which was present in the pull-down from bladder cancer samples only. The protein was identified with mass spectrometry as “V_1_-ATPase subunit D”, a component of the central stalk of the V_1_-ATPase vacuolar pump. The subunit D was confirmed in this complex by coimmunoprecipitation investigations and it was found to colocalize with the receptor. The subunit D was also shown to be overexpressed by Western blot, RT-PCR and immunofluorescence analyses. Immunoprecipitation and immunofluorescence also revealed that E5 oncoprotein was bound to the subunit D.

**Conclusion:**

For the first time, a tri-component complex composed of E5/PDGFβR/subunit D has been documented in vivo. Previous in vitro studies have shown that the BPV-2 E5 oncoprotein binds to the proteolipid c ring of the V_0_-ATPase sector. We suggest that the E5/PDGFβR/subunit D complex may perturb proteostasis, organelle and cytosol homeostasis, which can result in altered protein degradation and in autophagic responses.

## Introduction

Urinary bladder tumors are very rare in cattle, representing approximately 0.01% of all bovine malignancies [1]. However, these tumors occur endemically in adult cattle reared in hilly/mountain pasturelands rich in bracken fern (*Pteridium* spp.) [2]–[4]. The fern contains immunosuppressive, mutagenic, clastogenic and carcinogenic chemicals; therefore, it is believed to be the only higher plant naturally causing cancer in animals [5]. It has been suggested that toxic substances of fern have an important synergistic role in concert with infectious agents in bovine urinary bladder carcinogenesis [6], [7]. Furthermore, bovine papillomavirus type 2 (BPV-2) has a crucial role in bovine bladder carcinogenesis; BPV-2 DNA was found in 80% of naturally occurring cancers of the urinary bladder in cattle [4], [8]–[11]. Indeed, BPV-2 appears to be the most important infectious agent involved in bovine and bubaline urinary bladder carcinogenesis [9], [10], [12]–[18].

It has been suggested that BPV-2, a closely related serotype to BPV-1 [19], causes a latent infection of the urothelium, which can be activated by the chemical carcinogens of bracken fern ultimately resulting in bladder cancer [7].

The major transforming protein encoded by BPV-2 is the 44-amino acid polypeptide E5. Bovine and human papillomavirus E5 proteins appear to be localized in the membranes of the endoplasmic reticulum, the Golgi apparatus and in the plasma membrane of the host cell [20]. It has been shown that E5 oncoprotein of bovine papillomavirus is responsible for cell transformation via several pathways [21], [22] including the impairment of the V_0_-ATPase [23]. Furthermore, papillomavirus E5 protein is a powerful proteotoxic factor causing severe swelling and fragmentation of the Golgi apparatus and extensive vacuolization of the cytoplasm [24].

In vitro studies have revealed that BPV E5 oncoprotein can impair the vacuolar H+-ATPase proton pump as it is able to bind to its component, the cellular protein 16 k ductin/subunit c of the V_0_ domain [25]. This pump is essential for the acidification of the intracellular organelle compartments and may have an important role in protein sorting and processing [26]. Dysfunction of the H+-ATPase proton pump can result in the perturbation of acidification of the endomembrane components and the cytosol. Furthermore, it has been suggested that the 16 k protein allows E5 to bind to the PDGFβR, the activation of which has a central role in bovine bladder carcinogenesis [15], [18], [21], [26], [27].

Herein we present in vivo data showing that E5 binds to the subunit D of the V_1_-ATPase proton pump in naturally occurring urothelial bladder tumors in cattle.

## Materials and Methods

### Ethics Statement

In this study we did not perform any animal experiments. We collected the samples directly from public slaughterhouses; the animals were slaughtered following a mandatory clinical ante-mortem examination as required by European Union legislation.

### Tumor Samples

Fifteen bovine urothelial tumor samples and three normal (control) bladder samples were collected with the permission of the medical authorities in public and private slaughterhouses named “Macello Comunale” of Muro Lucano (PZ), “Barbara Rocco sas” of Simbario (VV), “Real Beef srl” of Flumeri (AV).

Bladder samples were routinely divided into several parts. Some parts were fixed in 10% buffered formalin for microscopic investigations. The remaining parts were immediately frozen in liquid nitrogen and stored at −80°C for subsequent biomolecular analysis.

### Histopathology

The tissues fixed in 10% buffered formalin were routinely paraffin embedded. Histologic diagnosis was assessed on 5-µm-thick hematoxylin-eosin (HE)–stained sections using morphologic criteria suggested in the recent report on the new histological classification of urothelial tumors of the urinary bladder of cattle [4].

### Immunohistochemistry

All samples were stained and sections of normal bovine urinary bladder mucosa were tested in parallel as controls. Briefly, sections were deparaffinized, and blocked for endogenous peroxidase in 0.3% H_2_O_2_ in methanol for 20 min. Antigen enhancement was performed by pretreating with microwave heating (twice for 5 min each at 750 W). The slides were washed three times with phosphate buffered saline (PBS, pH 7.4, 0.01 M). They were incubated for 1 h at room temperature with donkey serum (Santa Cruz Biotechnology Inc., CA, USA) diluted at 1 in 20 in PBS for the E5 detection and with protein block serum-free (DakoCytomation, Denmark) for V_1_-ATPase subunit D and pPDGFβR detection. The following primary antibodies were used: a purified polyclonal sheep anti-BPV-2 E5 (a kind gift by Dr. L. Nasir, Glasgow University), a monoclonal mouse anti-V_1_-ATPase subunit D (Santa Cruz Biotechnology Inc., CA, USA) and a polyclonal goat anti-pPDGFβR (phosphorylated at Tyr^770^) (Santa Cruz Biotechnology Inc., CA, USA). They were diluted at 1 in 5000, at 1 in 50, at 1 in 200 in phosphate buffered saline (PBS; pH 7.4, 0.01 M) for E5, V_1_-ATPase subunit D and pPDGFβR (phosphorylated at Tyr^770^) respectively and were applied overnight at room temperature in a humified chamber. All the slides were washed for 20 min with PBS. Then the slides were incubated for 30 min with a secondary donkey anti-sheep antibody (Santa Cruz Biotechnology Inc., CA, USA) diluted at 1 in 100 in PBS for the E5 detection and with appropriate biotinylated secondary antibody (labelled streptavidin-biotin (LSAB) Kit; DakoCytomation, Denmark) for V_1_-ATPase subunit D and pPDGFβR detection. Sections were washed three times with PBS and then incubated with streptavidin-conjugated to horseradish peroxidase (LSAB Kit, DakoCytomation, Denmark). Color development was obtained by treatment with diaminobenzidine (DakoCytomation, Denmark) for 5–20 min. Sections were counterstained with Mayer’s haematoxylin.

### Immunofluorescence

All samples were stained and sections of normal bovine urinary bladder mucosa were tested in parallel as control. For two-color immunofluorescence, sections were deparaffinized, rehydrated and heated in a microwave oven in citrate buffer (twice for 5 min each at 750 W) to allow antigen unmasking. Briefly, the sections were rinsed in PBS, pre-incubated for 1 h with normal donkey serum (diluted at 1 in 20) and then overlaid with the purified polyclonal sheep anti-BPV-2 E5 primary antibody diluted at 1 in 500 in phosphate buffered saline (PBS; pH 7.4, 0.01 M) (a kind gift by Dr. L. Nasir, Glasgow University) and the polyclonal goat anti-pPDGFβR (phosphorylated at Tyr^770^) primary antibodies (Santa Cruz Biotechnology Inc., CA, USA) diluted at 1 in 25 in phosphate buffered saline (PBS; pH 7.4, 0.01 M) were applied overnight at room temperature in a humified chamber.

Before the exposure to secondary antibodies, all the slides were washed for 20 min with PBS. A secondary antibody Alexa Fluor 488 donkey anti-sheep (Invitrogen, Molecular Probes) and a secondary antibody Alexa Fluor 546 donkey anti-goat (Invitrogen, Molecular Probes), diluted at 1 in 50 in PBS, were applied for 2 h at room temperature.

After washing 3 times with PBS, the slides were mounted under aqueous medium (Sigma-Aldrich, Milan, Italy).

An immunofluorescence staining was performed to detect V_1_-ATPase subunit D. The sections were treated as above, then the monoclonal mouse anti-V_1_-ATPase subunit D primary antibody (Santa Cruz Biotechnology Inc., CA, USA) diluted at 1 in 20 in phosphate buffered saline (PBS; pH 7.4, 0.01 M) was applied overnight at room temperature in a humid chamber. Before the exposure to secondary antibodies, all the slides were washed for 20 min with PBS. A secondary antibody Alexa Fluor 546 donkey anti-mouse (Invitrogen, Molecular Probes), diluted at 1 in 50 in PBS, was applied for 2 h at room temperature. After washing 3 times with PBS, the slides were mounted under aqueous medium (Sigma-Aldrich, Milan, Italy).

For two-color immunofluorescence staining of BPV-2 E5 and V_1_-ATPase subunit D, the sections were treated as above, then the polyclonal sheep anti-BPV-2 E5 (a kind gift by Dr. L. Nasir, Glasgow University) and the monoclonal mouse anti-V_1_-ATPase subunit D primary antibodies (Santa Cruz Biotechnology Inc., CA, USA) diluted respectively at 1 in 50 and 1 in 20 in phosphate buffered saline (PBS; pH 7.4, 0.01 M) were applied overnight at room temperature in a humid chamber. Before the exposure to secondary antibodies, all the slides were washed for 20 min with PBS. A secondary antibody Alexa Fluor 488 donkey anti-sheep (Invitrogen, Molecular Probes) and a secondary antibody Alexa Fluor 546 donkey anti-mouse (Invitrogen, Molecular Probes), diluted at 1 in 50 in PBS, were applied for 2 h at room temperature.

After washing 3 times with PBS, the slides were mounted under aqueous medium (Sigma-Aldrich, Milan, Italy).

For two-color immunofluorescence staining of V_1_-ATPase subunit D and pPDGFβR (phosphorylated at Tyr^770^), the sections were treated as above, then the monoclonal mouse anti-V_1_- ATPase subunit D primary antibody (Santa Cruz Biotechnology Inc., CA, USA) diluted at 1 in 20 in phosphate buffered saline (PBS; pH 7.4, 0.01 M) and the polyclonal goat anti-pPDGFβR (phosphorylated at Tyr^770^) primary antibody (Santa Cruz Biotechnology Inc., CA, USA) diluted at 1 in 25 in phosphate buffered saline (PBS; pH 7.4, 0.01 M) were applied overnight at room temperature in a humid chamber. Before the exposure to secondary antibodies, all the slides were washed for 20 min with PBS. A secondary antibody Alexa Fluor 546 donkey anti-mouse (Invitrogen, Molecular Probes) and a secondary antibody Alexa Fluor 488 donkey anti-goat (Invitrogen, Molecular Probes), diluted at 1 in 50 in PBS, were applied for 2 h at room temperature.

After washing 3 times with PBS, the slides were mounted under aqueous medium (Sigma-Aldrich, Milan, Italy).

For all immunofluorescence observations and photography, a laser scanning confocal microscope LSM-510 (Zeiss, Göttingen, Germany) was used.

### In-gel Digestion of IP Protein

Gel bands for mass spectrometric analysis were basically processed according to Shevchenko et al. [39]. Sliced gel pieces were washed with 100 mM NH_4_HCO_3_ and acetonitrile (1∶1, v/v) (buffer A). HPLC-grade acetonitrile was obtained from Sigma-Aldrich (St. Louis, MO). Proteins were in-gel reduced by 10 mM DTT, and subsequently alkylated with 20 mM iodoacetamide. After a washing step with buffer A, the gel pieces were dried in a vacuum centrifuge, and rehydrated at 4°C in digestion buffer (50 mM NH_4_HCO_3_, 5 mM CaCl_2_) containing 25 ng/µl trypsin. After overnight incubation, the peptides were extracted from the gel using three separate washings with a mixture of acetonitrile/water/formic acid 70/25/5 (v/v/v). The extracts were combined and dried down in a vacuum centrifuge. The lyophilized digest was reconstituted in 30 µl of loading pump solvent (see nano LC-MS/MS Section). Ten µl of the solution were then injected for nano LC-MS/MS analysis.

### Nano LC-MS/MS and Database Search

Chromatography was performed using an Ultimate nanoscale liquid chromatography (nano LC) system from Dionex (Sunnyvale, CA). The analytical nano LC column used was an in-house packed 75 µm i.d., 40 cm long Integra Frit™ column obtained from New Objective (Cambridge, MA), filled with 4 µm C_12_ silica particles Jupiter Proteo from Phenomenex (Torrence, CA). Ten µL of the peptide mixture were loaded onto an in-house packed 150 µm i.d., 3 cm long Integra Frit™ (New Objective) trapping column (packing bed length 1 cm) at 12 µL/min of loading pump solvent, consisting of H_2_O/acetonitrile/trifluoroacetic acid (TFA) 97.95∶2:0.05 (v/v/v). After 2 minutes washing, the trapping column was switched on-line to the analytical column, and gradient separation started at 200 nL/min.

A binary gradient was used for peptide elution. Mobile phase A was H_2_O/acetonitrile/formic acid/TFA 97.9∶2:0.09∶0.01 (v/v/v/v); mobile phase B was H_2_O/acetonitrile/formic acid/TFA 29.9∶70:0.09∶0.01 (v/v/v/v). Gradient was from 5 to 45% B in 60 minutes at 200 nL/min flow rate. After 10 minutes at 95% B, the column was re-equilibrated at 5% B for 30 minutes before the following injection.

MS detection was performed on a QSTAR XL hybrid mass spectrometer from Applied Biosystems (Foster City, CA) operating in positive ion mode, with nanoelectrospray (nESI) potential at 1800 V, curtain gas at 15 units, CAD gas at 3 units. nESI ionization was achieved via distal coated Pico Tips™ 20 µm ID, 10 µm tip ID (New Objective). Information-dependent acquisition (IDA) was performed by selecting the two most abundant peaks for MS/MS analysis after a full TOF-MS scan from 400 to 1600 *m/z* lasting 4 seconds. Both MS/MS analyses were performed in enhanced mode (3 seconds/scan). Threshold value for peak selection for MS/MS was 20 counts.

Data were searched on the Mascot search engine (www.matrixscience.com) against the Swiss Prot database using the following parameters: MS tolerance 10 ppm; MS/MS tolerance 0.3 Da; fixed modifications carbamidomethyl cysteine; enzyme trypsin; max. missed cleavages 1; taxonomy other mammalia.

Protein hits based on two successful peptide identifications were considered valid. Protein hits based on a single peptide identification with Mascot score higher than the significance level (>14) were retained after manual validation.

### BPV-2 E5 and PDGFβR Immunoprecipitation

Tissues were lysed in ice-cold buffer containing 50 mM Tris-HCl (pH 7.5), 1% (v/v) Triton X-100, 150 mM NaCl, 2 mM PMSF, 1.7 mg/ml Aprotinin, 50 mM NaF, and 1 mM sodium orthovanadate. The protein concentration was measured using the Bradford assay (Bio-Rad Laboratories, Milan, Italy). Proteins (1000 µg) were immunoprecipitated using 2 µg of anti- E5 antibody (a kind gift of Dr L. Nasir, Glasgow University) or anti-PDGFβR antibody (Santa Cruz Biotechnology, CA, USA) and 30 µl of Protein A/G-Plus Agarose (Santa Cruz Biotechnology, CA, USA). Immunoprecipitates were washed four times in complete lysis buffer (above), finally heated in 1X Laemmli sample buffer at 100°C for 10 minutes. Immunoprecipitates were separated on polyacrylamide gels and transferred to nitrocellulose filter membranes (Ge Healthcare Life Sciences, Chalfont St Giles, UK) for 16 h at 30 mA in 192 mM glycine/25 mM Tris-HCl (pH 7.5)/10% methanol. Membranes were blocked for 1 h at room temperature in 5% nonfat dry milk, incubated with anti-E5 antibody, anti-PDGFβR, anti-pPDGFβR (phosphorylated at Tyr^770^), anti-V_1_-ATPase subunit D (Santa Cruz Biotechnology, CA, USA), and anti V_0_-ATPase c subunit (Cosmo Bio CO, Japan) overnight at 4°C. After three washes in Tris-buffered saline, membranes were incubated with rabbit anti-sheep IgG-horseradish peroxidase (HRP) (Santa Cruz Biotechnology, CA, USA) or with goat anti-rabbit or anti-mouse IgG (Bio-Rad Laboratories, Milan, Italy) for 60 min at room temperature. Proteins were visualized by enhanced chemiluminescence system (Western Blotting Luminol Reagent, Santa Cruz Biotechnology, CA, USA) and ChemiDoc XRS Plus (Bio-Rad Laboratories, Milan, Italy). Images were acquired with Image Lab Software version 2.0.1 (Bio-Rad Laboratories, Milan, Italy).

### BPV-2 DNA Detection and Sequencing

DNA was extracted from urinary bladder samples from frozen pathological and normal (control) bladder samples using the DNeasy Tissue kit (Qiagen, Milan, Italy) according to the manufacturer’s protocol. All the samples were lysed using proteinase K. Lysates were loaded onto DNeasy spin columns. After two washing steps pure DNA was eluted with low salt buffer. To amplify the entire BPV-2 genome, the purified DNA was subjected to multiply primed rolling-circle amplification using a reaction mixture containing 20 ng sample DNA, 12.5 µM of each primer, 4 mM dNTPs and 10 U phi 29 DNA polymerase (Fermentas, Milan, Italy). The resulting linear dsDNA product was purified using MinElute PCR Purification kit (Qiagen, Milan, Italy). For the detection of BPV-2 DNA, specific primers for a 503 bp DNA amplicon encompassing the BPV-2 E5-L2 ORF sequence (nt 3723–4225) were designed by Primer BLAST software (forward, 5′-TCAGGCACAGATCTTGATCA-3′; reverse, 5′-TCATAGACATTTGCACGTT-3′). To evaluate the adequacy of the DNA samples, a control PCR for bovine β-actin sequence was performed using a set of primers designed by Primer BLAST software (forward, 5′-GAGCGTGGCTACAGCTTCAC-3′; reverse, 5′-CATTGCCGATGGTGATGA-3′). Aliquots 50–100 ng of purified DNA were amplified in 25 µl of reaction mixture containing 2 mM MgCl_2_, 200 µM each dNTP, 480 nM of each primer and 2.5 U of AmpliTaq Gold DNA Polymerase (Applied Biosystems, Monza, Italy). The reaction was carried out in a thermocycler (Veriti, Applied Biosystems, Monza, Italy) with an initial denaturation step of 3 min. Then, 35 cycles of amplification were carried out with a denaturation step at 94°C for 40 sec, an annealing step at 60°C, 30 sec, for β-actin or at 50°C,40 sec, for BPV-2, and an extension step at 72°C for 1 min. A final extension step at 72°C for 7 min was performed in each PCR assay. Detection of the amplified products was carried out by electrophoresis on ethidium bromide-stained agarose gel. In each experiment, a blank sample consisting of reaction mixture without DNA and a positive sample consisting of cloned BPV-2 (a kind gift by Dr. A. Venuti) were included. The amplified DNA was subjected to direct sequencing in an automated apparatus (ABI Prism 3100 Genetic Analyzer; Applied Biosystems, Monza, Italy).

### Western Blot Analysis

Healthy and diseased bladders were solubilized at 4°C in lysis buffer containing 50 mM Tris-HCl pH 7.5, 150 mM NaCl, 1% Triton X-100. Immediately prior to use, the following reagents were added: 1 mM DTT, 2 mM PMSF, 1.7 mg/ml Aprotinin, 25 mM NaF, 1 mM Na_3_VO_4_ (Sigma-Aldrich, Milan, Italy).

Lysates were clarified at 500×g for 20 min. The protein concentration was measured using the Bradford assay (Bio-Rad Laboratories, Milan, Italy). For Western blotting, 50 µg of lysate proteins were heated at 100°C in 4X premixed Laemmli sample buffer (Bio-Rad Laboratories, Milan, Italy). Proteins were subjected to sodium dodecyl sulfate–polyacrylamide gel electrophoresis (SDS–PAGE) under reducing conditions.

After electrophoresis, proteins were transferred onto nitrocellulose filter membranes (GE Healthcare Life Sciences, Chalfont St Giles, UK) for 1 h at 10 V in 192 mM glycine/25 mM Tris-HCl (pH 7.5)/10% methanol using a Trans-Blot SD Semi Dry cell (Bio-Rad Laboratories, Milan, Italy) according to the manufacturer’s instructions. The membranes were blocked with 5% non-fat dry milk in Tris-buffered saline (TBS, pH 7.5) for 1 h at room temperature, washed with TBS-0.1% Tween. Then, filters were probed both with anti-PDGFβR, anti-pPDGFβR (phosphorylated at Tyr^770^), and anti-V_1_-ATPase subunit D antibody (Santa Cruz Biotechnology, CA, USA) for an overnight incubation at 4°C. After three washes in Tris-buffered saline, membranes were incubated with horseradish peroxidase-conjugated anti-rabbit IgG or anti-mouse IgG (Bio-Rad Laboratories, Milan, Italy) and anti-goat IgG (Santa Cruz Biotechnology, CA, USA), for 1 h at room temperature. After appropriate washing steps, protein detection and image acquisition were performed as above reported.

### RNA Extraction

Total RNA was extracted from urinary bladders of cows using the RNeasy Mini Kit (Qiagen, Milan, Italy), according to the manufacturer’s instructions. The RNA quality was determined by agarose gel electrophoresis and ultraviolet spectrophotometer analysis. The RNA was treated with RNase-free DNase I Fermentas Life Sciences (Dasit, Milan, Italy) to remove potential DNA contamination.

### cDNA Synthesis and Real Time-PCR Analysis (RT-PCR) for V_1_-ATPase Subunit D

For Real Time-PCR analysis, 500 ng RNA were reverse-transcribed using the iScript cDNA Synthesis Kit (Bio-Rad Laboratories, Milan, Italy) and the reaction was incubated at 25°C for 5 min, 42°C for 30 min, 85°C for 5 min, and then kept at 4°C for 5 min. Real Time reactions were performed using SsoFast EvaGreen Supermix (Bio-Rad Laboratories, Milan, Italy). For the detection of V_1_-ATPase subunit D specific primers (forward primer, 5′-AAGACTCAGTGGCTGGGTTG -3′; reverse primer, 5′-AGGTTTCGACCTGTCTGTGC-3′) were used. All reactions were performed in triplicate and β-actin was used as the internal standard (forward primer 5′- TAGCACAGGCCTCTCGCCTTCG-3′; reverse primer 5′- GCACATGCCGGAGCCGTTGT-3′).

## Results

### Microscopical Pattern of the Tumors

Histological patterns of urothelial tumors of the urinary bladder of cattle were consistent with the diagnosis of papillary urothelial neoplasm of low malignant potential (PUNLMP) (two cases), papillary and invasive urothelial cancers (nine and four cases, respectively).

### PCR Analysis and BPV-2 Sequencing

**Figure 1:**
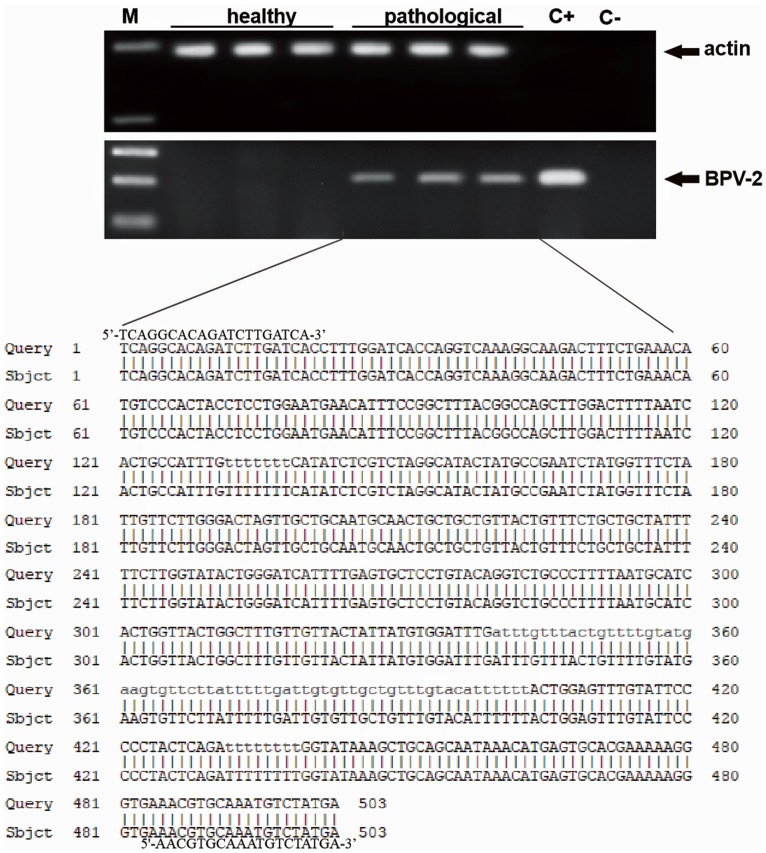
PCR amplification and sequencing of BPV-2 DNA. Lane M, Molecular mass marker (1 kb DNA Ladder, Microtech). Lanes 1–3: Three normal (control) samples from healthy cows. Lanes 4–6: three representative tumor samples. Lane C+: positive control containing a cloned BPV-2 DNA. Lane C-. negative control (no DNA added). The lower part of the figure shows 100% identity between the sequence of the amplicons in lanes 4–6 and the sequence of BPV-2 deposited in GenBank (M20219.1).

PCR yielded BPV-2 DNA fragments of anticipated size (503 bp) for all neoplastic lesions. No BPV-2 DNA was detected in normal (control) bladder samples (Figure 1). The presence of BPV-2 DNA was also confirmed by sequencing (Figure 1) according to BPV-2 sequence M20219.1.

### Immunoprecipitation and Immunohistochemistry for BPV-2 E5 Protein

**Figure 2:**
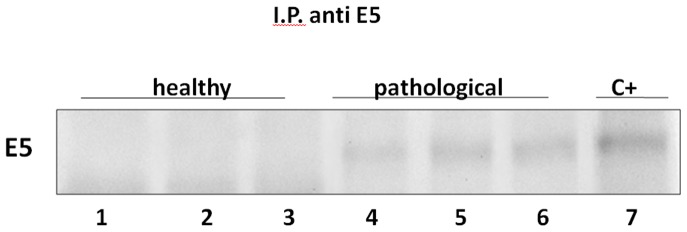
BPV-2 E5 immunoprecipitation. The presence of E5 protein detected by immunoprecipitation. a) Lanes 1–3: urinary bladders from healthy cows. Lanes 4–6: three representative urothelial tumors of the urinary bladder in cows. Lanes 7: positive control (bovine placenta infected with BPV-2).

**Figure 3:**
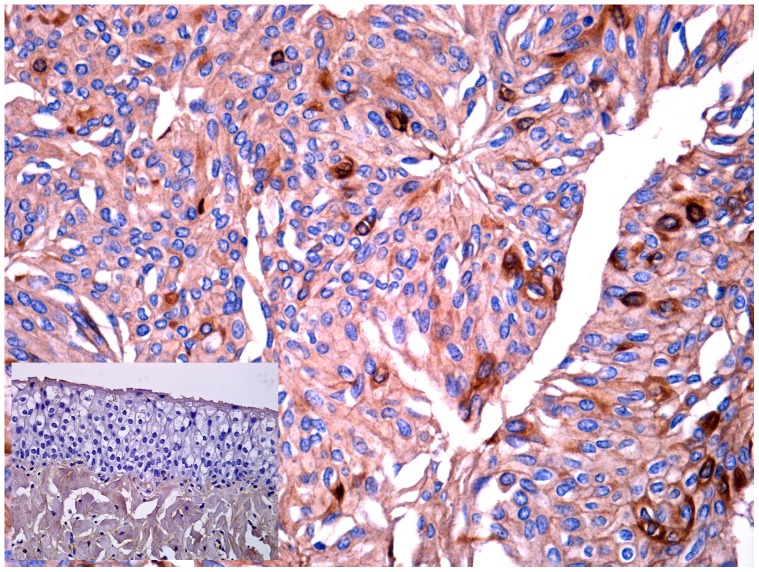
BPV-2 E5 immunohistochemistry. Urothelial carcinoma. Immunohistochemical detection of cytoplasmic E5 in neoplastic urothelial cells. E5-expressing cancer cells are scattered both in basal and suprabasal layers. Magnification, ×550. Insert: normal (control) urothelium from healthy cows. Magnification, ×550.

The expression of E5 was detected by immunoprecipitation and immunohistochemistry in tumor samples. E5 immunoreactivity was evident in cells located in basal and suprabasal urothelial layers (Figure 2 and 3).

### Coimmunoprecipitation and Colocalization of E5 Oncoprotein with PDGFβR

**Figure 4:**
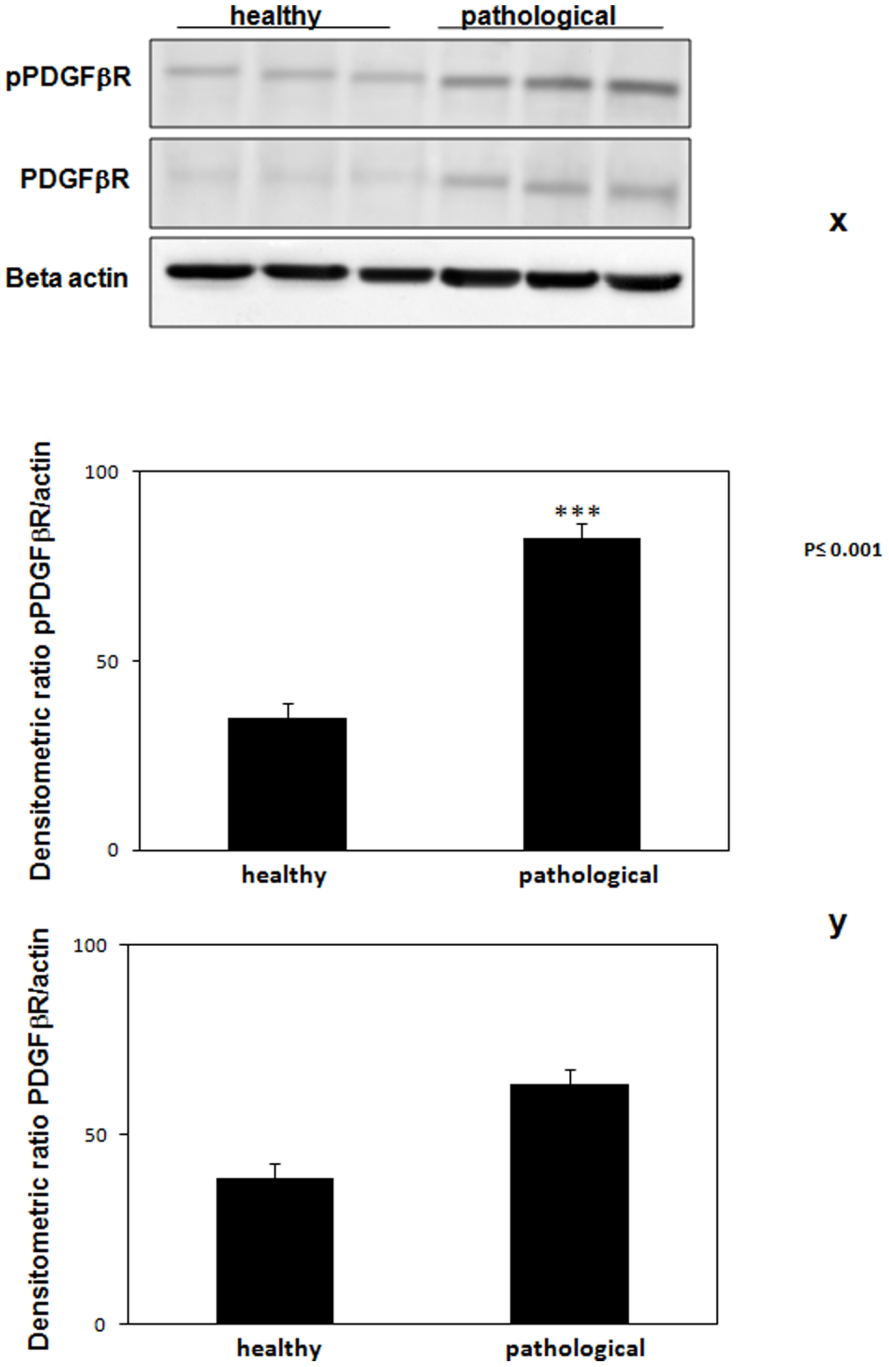
Total and phosphorylated (activated) PDGFβR expression. (x) Total protein extracts from tissue lysates were generated and used in Western blot analysis with an antibody specific for total PDGFβR and a phosphospecific PDGFβR antibody that recognized pPDGFβR phosphorylated at Tyr770. Lanes 1–3: urinary bladder from healthy animals. Lanes 4–6: representative neoplastic tissues from three cows with papillomavirus-associated tumors of the urinary bladder. Actin protein levels were detected to ensure equal protein loading. (y) Quantitative densitometric analysis of the filters was performed with Image Lab software (ChemiDoc; Bio-Rad Laboratories) and significance determined by the Student T-test (***, p<0.001).

**Figure 5:**
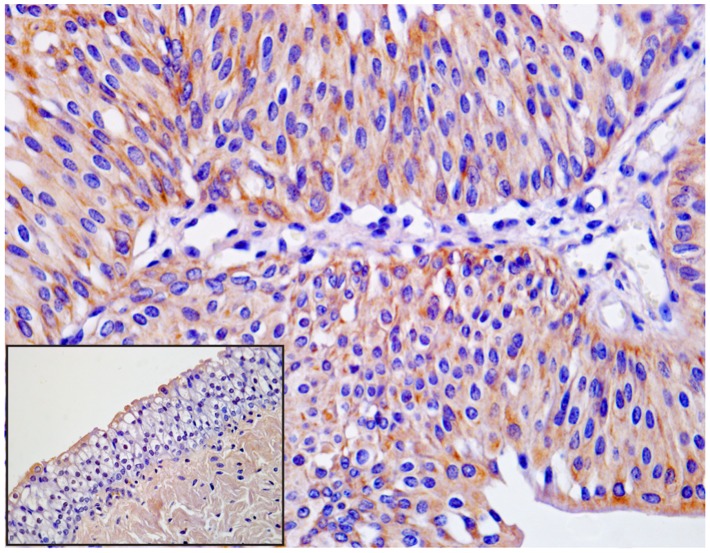
pPDGFβR immunohistochemistry. Urothelial carcinoma. Immunohistochemical detection of phosphorylated PDGFβR in neoplastic urothelial cells and in normal (control) urothelial cells as shown in the insert. Magnification, ×550.

Previous in vivo studies have shown that E5 binds to the activated (phosphorylated) form of PDGFβR in bladder tumors [18], [21], [27]. Indeed, PDGFβR appeared to be constitutively expressed and its phosphorylation was increased in the tumor samples compared to the healthy ones as detected in total lysates (Figure 4). The activation of the phosphorylated PDGFβR was also documented by immunohistochemical investigations (Figure 5).

**Figure 6:**
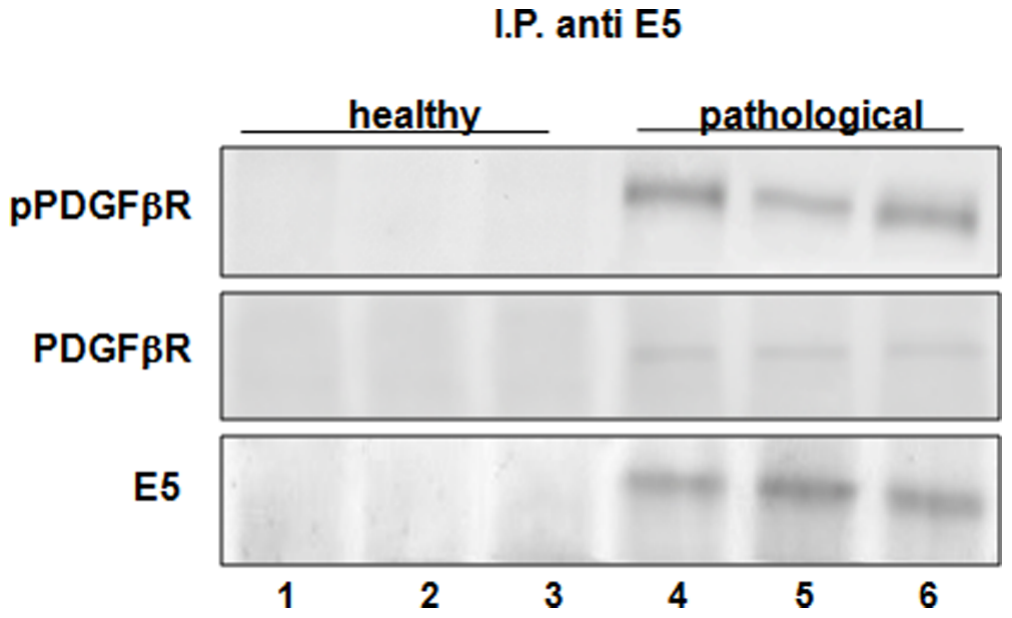
BPV-2 E5 and PDGFßR co-immunoprecipitation. The presence of phosphorylated and total PDGFßR was detected in E5 immunoprecipitates. Lanes 1–3: urinary bladder from healthy cows. Lanes 4–6: cancer tissue from three cows with papillomavirus-associated tumors of the urinary bladder.

**Figure 7:**
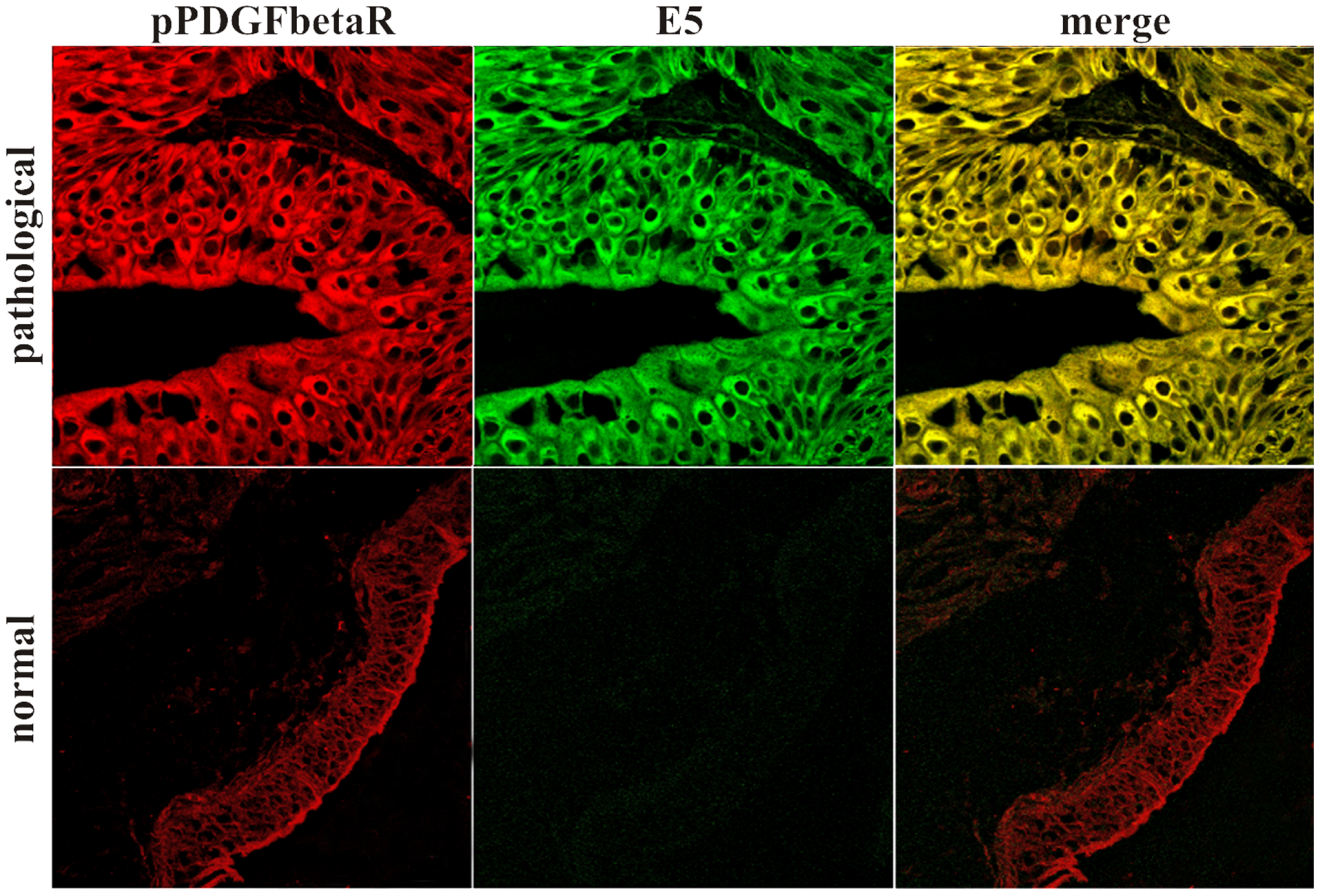
E5 and pPDGFβR colocalization. Urothelial carcinoma. Immunofluorescence detection of E5 and pPDGFβR and their colocalization (yellow in the merged images) in urothelial cancer cells (upper) vs normal (control) urothelial cells (lower). Magnification ×550.

A coimmunoprecipitation experiment using an anti-E5 antibody was carried out and PDGFβR and pPDGFβR were detected by Western blot in the immunoprecipitates (Figure 6). Morphologically, the E5/pPDGFβR complex was shown by confocal microscopy: E5 and the activated form of the PDGFβR appeared to co-localize as judged by the yellow fluorescence of the merged images (Figure 7). Normal urothelium yielded no E5 signal.

### PDGFβR Binds to the Component D of V_1_-ATPase: Proteomic Analysis

In vitro, PDGFβR can bind to the proteolipid ring of V_0_ sector of the proton pump in absence of E5, it has been suggested that E5 binds to the PDGFβR via the subunit c of the ring**.** However, these data have been obtained for medium-adapted cultured cells and hence may not reflect the authentic in vivo situation, for which no information is available thus far [26].

Accordingly, we investigated whether a similar complex could take place in vivo and performed immunoprecipitation of PDGFβR on bladder carcinoma tissue and on control normal bladder tissue from healthy cattle.

**Figure 8:**
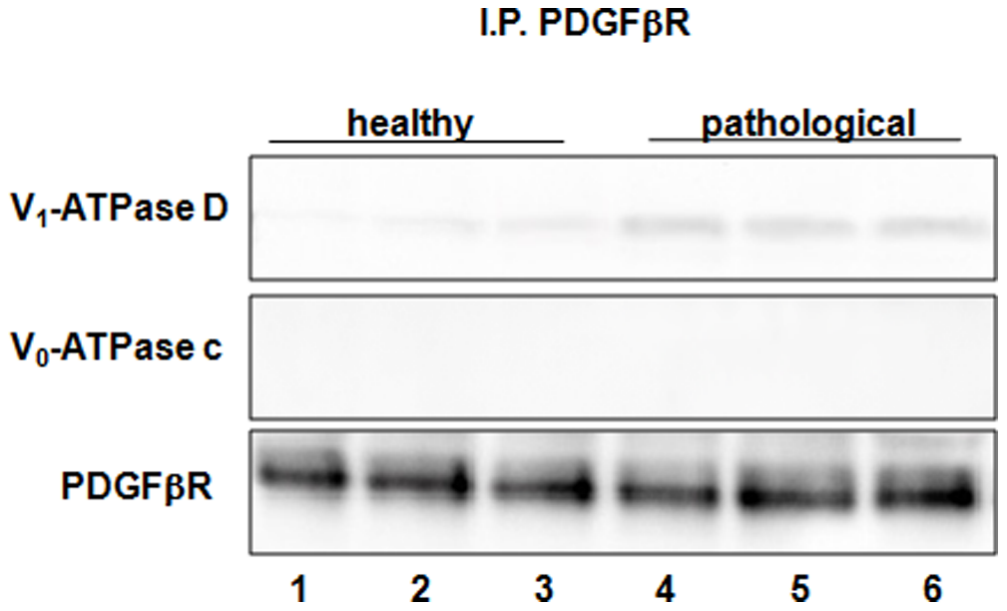
PDGFßR and V_1_-ATPase co-immunoprecipitation. PDGFβR interaction with V_1_-ATPase subunit D is increased in immunoprecipitates derived from pathological tissues. V_0_-ATPase c subunit does not co-immunoprecipitate with PDGFβR. Lanes 1–3: urinary bladder from healthy cows. Lanes 4–6: cancer tissue from three cows with papillomavirus-associated tumors of the urinary bladder.

**Figure 9:**
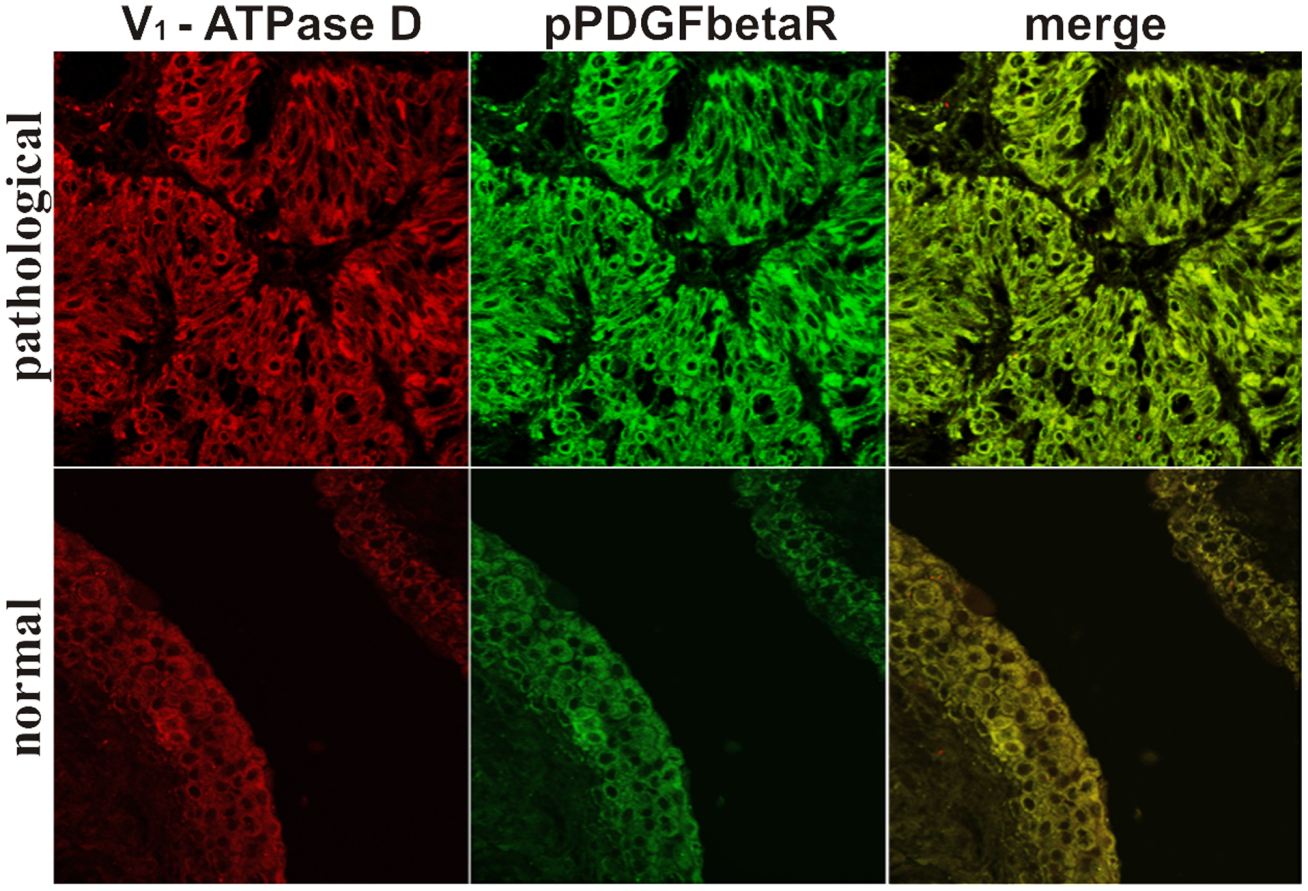
V_1_-ATPase subunit D and pPDGFβR colocalization. Urothelial carcinoma. Immunofluorescence detection of the V_1_-ATPase subunit D and the pPDGFβR and their colocalization signal (yellow in the merged image). Magnification, ×550.

Proteins contained in the two PDGFβR pull-downs from bladder tumor samples and from normal bladder tissue, were separated with SDS-PAGE and detected with Coomassie staining. Differential analysis between the protein bands contained in the two immunoprecipitates revealed a single band present in the PDGFβR pull-down from bladder tumor samples only. The differential band was excised and in-gel digested with trypsin. The peptides were injected for nano LC-MS/MS analysis. Database search of MS/MS spectra allowed the identification of the protein “V1-ATPase subunit D” (Table 1). Because the protein has been identified by a single peptide hit having a low Mascot score, this result needed to be validated by further investigations. For this purpose, we performed Western blot analysis using an anti-subunit D antibody on the PDGFβR immunoprecipitates. This allowed us to detect the presence of the subunit D (Figure 8). We also performed Western blot analysis using an anti-subunit c antibody on these immunoprecipitates as the PDGFβR/subunit c complex was documented in *in vitro* studies [25]. We did not detect the subunit c of the V_0_-ATPase domain (Figure 8). The complex pPDGFβR/subunit D of the V_1_-ATPase was shown by immunofluorescence studies as the two proteins appeared to colocalize by confocal microscopy (Figure 9).

**Table 1:** Protein identified by nanoLC-MS/MS analysis of IP band.

Accession	Protein Description	N.Peptides	Peptide Information	Protein Theor.Mw
VATD_BOVIN	V_1_-type proton ATPase subunit D	1	Sequence: IEIFPSR Mascot score: 14Theoretical Mw: 860.48 ExperimentalMw: 860.47	28 kDa

Peptide sequence, and relative protein entry, identified in IP band. Minimum Mascot score for confident identification was 14. MS/MS spectrum was manually validated. Contaminants such as human keratins and trypsin were excluded from the list.

### Western Blot Analysis of the Subunit D

**Figure 10:**
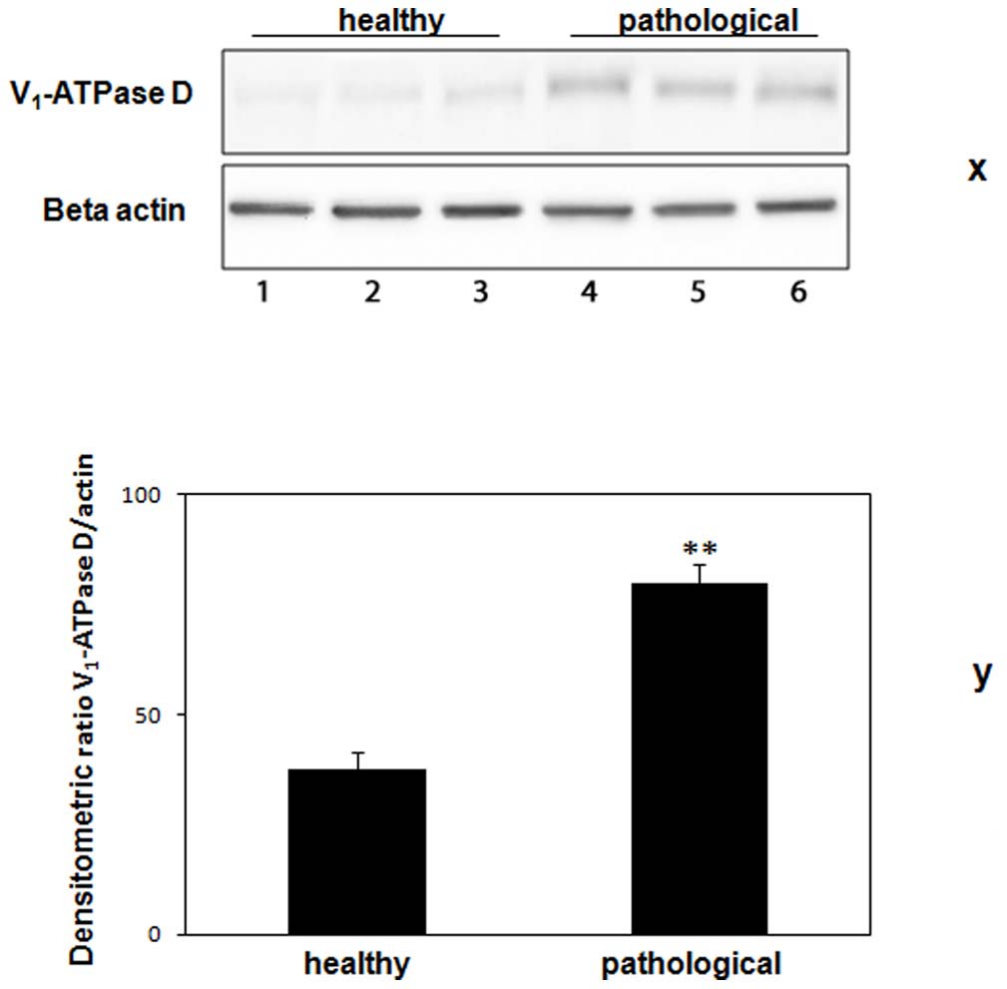
V_1_-ATPase subunit D expression. (x) Western blot analysis showing overexpression of V_1_-ATPase subunit D. Lanes 1–3: urinary bladder from healthy animals. Lanes 4–6: neoplastic tissue from representative three cows with papillomavirus-associated tumors of the urinary bladder. Actin protein levels were detected to ensure equal protein loading. (y) Quantitative densitometric analysis of the filters was performed with Image Lab software (ChemiDoc; Bio-Rad Laboratories) and significance determined by the Student T-test (**, p<0.01).

**Figure 11:**
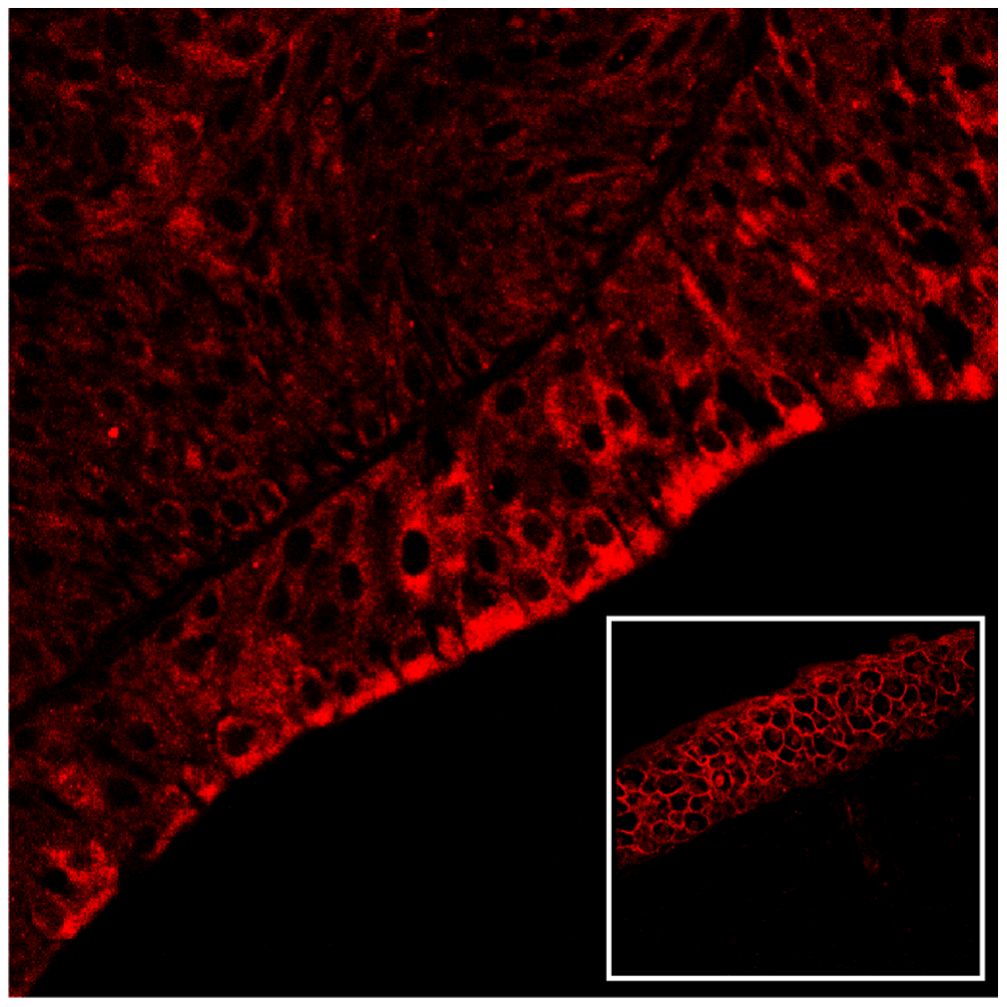
V_1_-ATPase subunit D immunofluorescence. Urothelial carcinoma. The overexpression of the subunit D of the **V_1_-**domain is also detected by immunofluorescence. The subunit D appears to be overexpressed both in the membrane and in the cytoplasm of urothelial cancer cells compared to urothelial normal cells (insert). Magnification, ×550.

**Figure 12:**
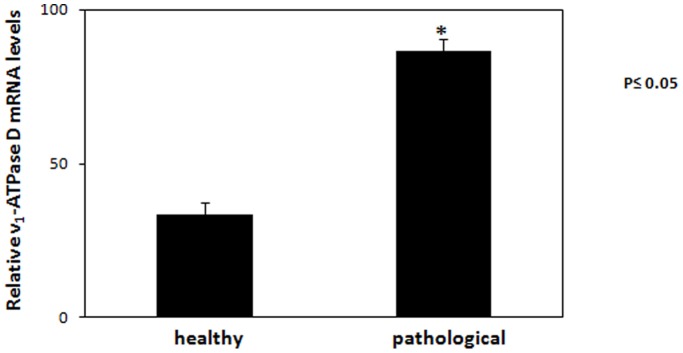
RT PCR for subunit D. The relative expression levels of V_1_-ATPase subunit D in neoplastic tissues. V_1_-ATPase subunit D mRNA levels were determined by qRT-PCR. Relative mRNA levels, calculated using the ΔΔC_T_ method, represent fold changes in comparison to urinary bladder samples from healthy cows. All values were normalized to the internal control β-actin. Results represent the means and standard deviations of three independent experiments performed in triplicate. (*, p<0.05, vs urinary bladder from healthy cows).

**Figure 13:**
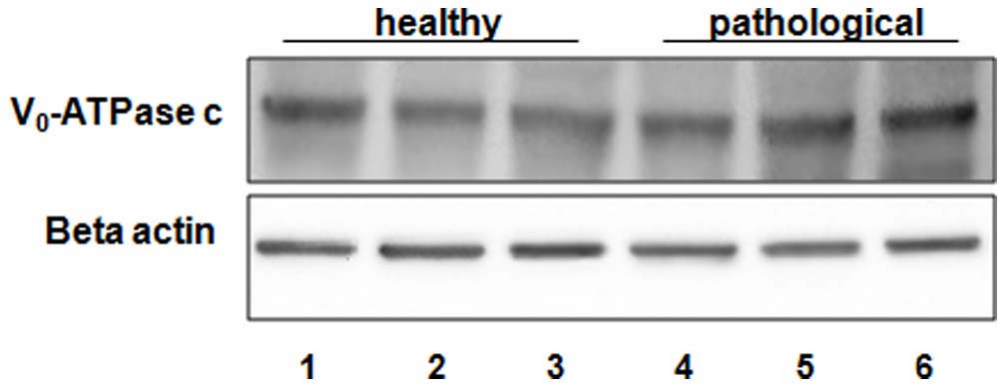
V_0_-ATPase c subunit expression. Western blot analysis showing similar expression levels of V_0_-ATPase c subunit among all the samples. Lanes 1–3: urinary bladder from healthy animals. Lanes 4–6: neoplastic tissue from representative three cows with papillomavirus-associated tumors of the urinary bladder. Actin protein levels were detected to ensure equal protein loading.

We performed immunoblotting to reveal the total level of the subunit D of the V_1_-domain. Overexpression of subunit D could be shown by immunoblotting (Figure 10) and morphologically documented by immunofluorescence (Figure 11). Furthermore, a statistically significant increase of the transcripts of this subunit was also shown by RT-PCR (Figure 12). Normal levels of the constitutively expressed subunit c were detected by Western blot both in healthy and tumor samples (Figure 13).

### Coimmunoprecipitation and Colocalization of E5 Oncoprotein with the Subunit D of the V1-ATPase

**Figure 14:**
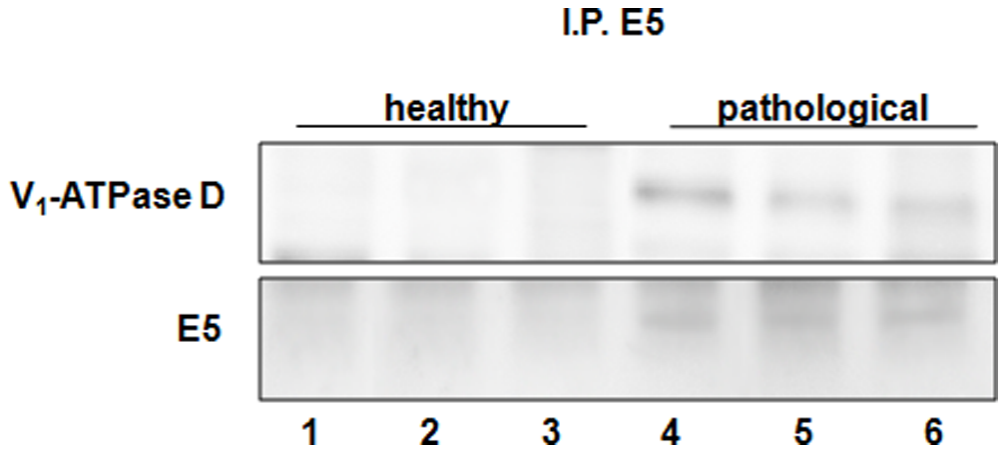
BPV-2 E5 and V_1_-ATPase subunit D co-immunoprecipitation. The presence of V_1_-ATPase subunit D was detected in E5 immunoprecipitates. Lanes 1–3: urinary bladder from healthy cows. Lanes 4–6: neoplastic tissue from three cows with papillomavirus-associated tumors of the urinary bladder.

**Figure 15:**
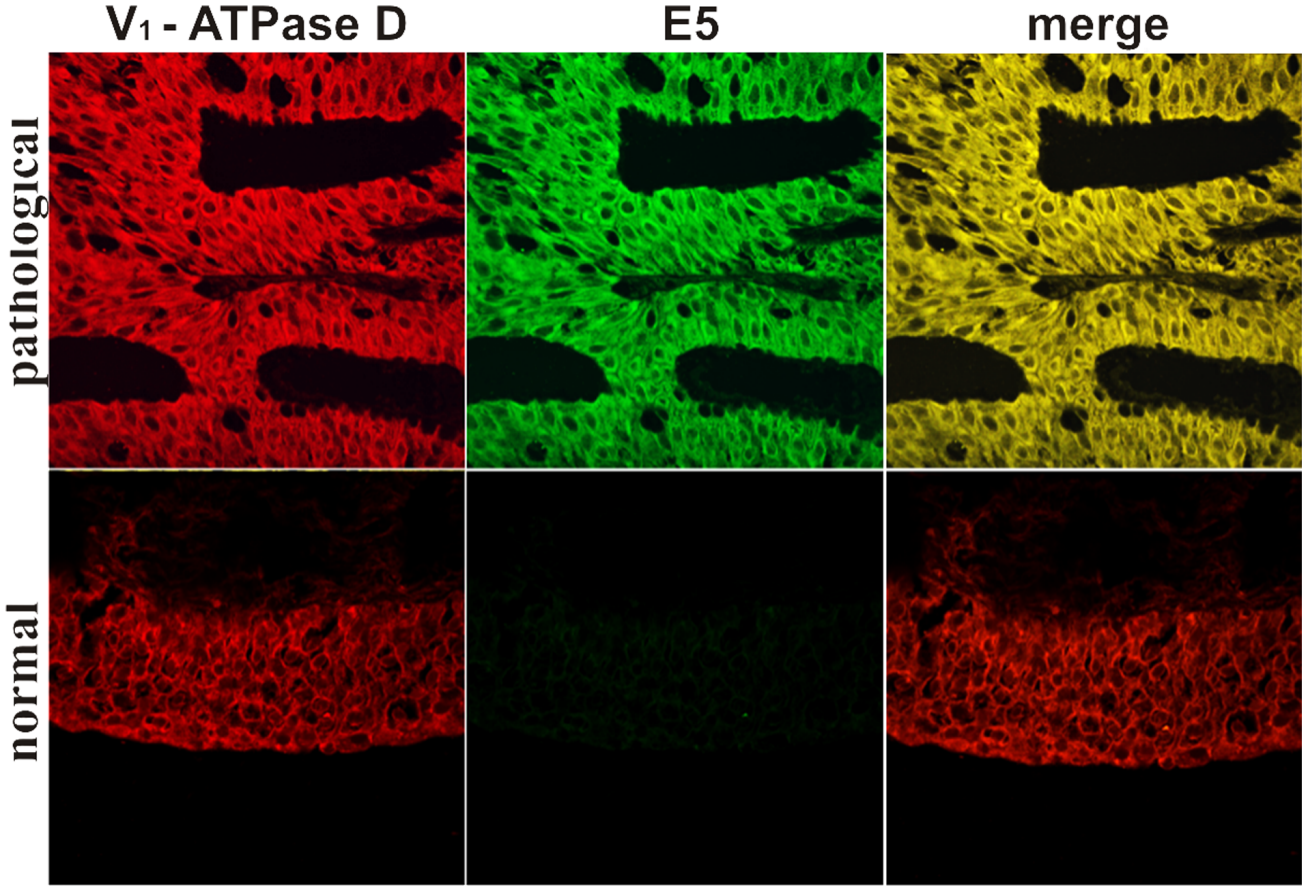
V_1_-ATPase subunit D and BPV-2 E5 colocalization. Urothelial carcinoma. Immunofluorescence detection of E5 and the subunit D. The proteins appear to colocalize (yellow in the merged image).

Using Western blot, we detected the subunit D of the V_1_-domain of the proton pump in E5 immunoprecipitates (Figure 14). Morphologically, this complex was demonstrated by confocal microscopy as E5 and subunit D appeared to colocalize (Figure 15). Normal urothelium yielded no E5 signal.

**Figure 16:**
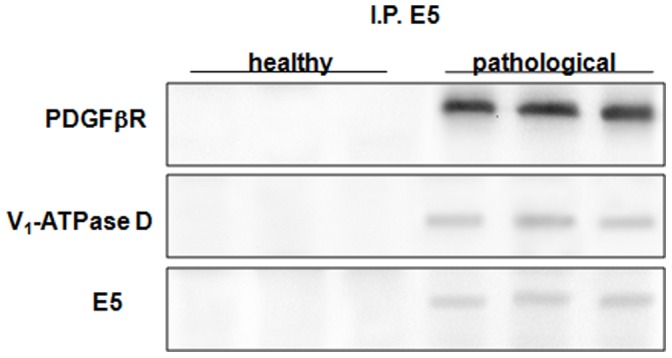
BPV-2 E5, PDGFßR and V_1_-ATPase D subunit co-immunoprecipitation. The presence of PDGFßR and V_1_-ATPase D subunit was detected in E5 immunoprecipitates. Lanes 1–3: urinary bladder from healthy cows. Lanes 4–6: tissue from three cows with papillomavirus-associated tumors of the urinary bladder.

Ultimately, both pPDGFβR and subunit D of the V_1_-ATPase were found by Western blot in E5 immunoprecipitates (Figure 16).

## Discussion

Our results indicate, for the first time, that a ternary complex composed of BPV-2 E5 oncoprotein/PDGFβR/subunit D of V_1_-ATPase is present in urothelial cells of naturally occurring tumors of the bovine urinary bladder.

It has been shown that bovine papillomavirus E5 interacts with the subunit c of the V_0_ domain in cultured cells [25]. As PDGFβR can bind to the subunit c in the absence of E5, it has been suggested that E5 binds to PDGFβR via its association with subunit of V_0_-ATPase proton pump [26].

Our in vivo findings appear to be different from previous in vitro results and show that the complex composed of E5 and the activated form of PDGFβR is associated with the overexpressed subunit D of the V_1_ domain, which catalyzes ATP hydrolysis, but not with the subunit c of the V_0_ domain responsible for H^+^ translocation. Furthermore, the subunit c appeared to be constitutively expressed, as normal levels of expression were shown to occur both in normal and neoplastic tissues.

It has been suggested that cell perturbation resulting from binding of the subunit c of the proton pump and E5 oncoprotein is responsible for Golgi alkalinization which, in turn, leads to the activation of Golgi-associated Src molecules. Indeed, Golgi alkalinization and c-Src are involved in a common mechanism leading to E5-dependent NIH3T3 cell transformation [28].

The subunit D of the V_1_ complex belongs to a central rotor stalk, which is composed also of F subunit. These proteins are bound directly to the subunit d of the V_0_ sector that links to the c ring. Therefore, the central stalk (DFd complex) connects the V_1_ and V_0_ domains. It serves as a rotor that couples the energy that is released from the hydrolysis of ATP to the rotation of the proteolipid c ring of the V_0_-ATPase pump and causes active transport of protons thus regulating the pH (acidification) of intracellular organelles and cytosol [29]–[31]. The controlled pH of the intracellular compartments is crucial for many biological processes, including membrane trafficking and protein degradation. It is conceivable that in naturally occurring bovine bladder cancers, the complex E5/PDGFβR/subunit D could have an important role in perturbing proteostasis network as well as organelle and cytosol homeostasis. It is worthwhile noting that in bovine urothelial tumors we detected an overexpression of some of the most important markers of proteostasis stress such as heat shock proteins (HSPs) [32]. The latter are known to act as molecular chaperones to restore protein homeostasis [33], [34]. Furthermore, we found an overexpression of the co-chaperone BAG3 (Bcl-2 associated athanogene 3), which depends on an altered degradation of the protein rather than the upregulation of gene transcription [35]. It is worth remembering that BAG3 protein degradation occurs via proteasome system only.

Cytosolic pH has been identified as a novel regulator that mediates the formation of proteasome storage granule (PSGs) and other protein aggregates. The regulation of proper partitioning of the proteasome into PSGs is essential for maintaining the correct level of the proteasome in the cytosol. It has been shown that the impaired ability of V-ATPase to regulate intracellular pH affects the kinetics of the PSG formation [30].

In our cases, the impairment of the vacuolar pump induced by E5 oncoprotein can be responsible for a proteasomal dysfunction resulting in a reduced clearance of specific protein such as proteasome-degraded BAG3 protein, known to be involved in a plethora of biological processes including the key role in mitigating the proteotoxicity via selective autophagy [36]. It has been shown that autophagy is activated when the proteasome function is reduced, thus constituting a strong functional link between autophagy and proteasome systems [37]. Our findings are consistent with several in vitro studies showing that the impairment of V-ATPase can induce autophagic responses and increase the formation of autophagosomes thus the autophagy represents a mechanism to overcome alteration of pH homeostasis mediated by proton pump perturbation [38].

Finally, the selective autophagy occurring in bovine urothelial tumor cells transformed by E5 oncoprotein that we have been studying (Roperto, unpublished data) seems to strengthen this suggestion and the emerging concept that the molecular chaperones, the ubiquitin-proteasome system (UPS) and the autophagy machinery are central elements of the proteostasis network in which the V-ATPase proton pump is also involved.
